# The X-Rays

**Published:** 1912-04-20

**Authors:** Alfred C. Norman

**Affiliations:** House Surgeon at Sunderland and Durham County Eye Infirmary, Sunderland.


### ELECTRICITY IN MODERN MEDICINE.*

### X.—The X-Rays.

By ALFRED C. NORMAN, M.D. Edin., House Surgeon at Sunderland and Durham County Eye Infirmary, Sunderland.

The induction coil we are considering is to be used in conjunction with a mercury interrupter; a Walter switch is therefore unnecessary. With a mercury interrupter the coil gives best results when there is a high degree of self-induction in the primary circuit; the makers should therefore be asked to make arrangements for connecting all four layers of the primary winding permanently in series. This is done by insulated wires which pass from end to end of the primary coil in such a way that the ending of the first layer, terminal E1, is connected with the beginning of the second layer, terminal A2, and so on for the four layers. These external connecting wires are best kept out of harm's way by passing them through or underneath the wood base of the coil, and the makers should number their free ends, so that the user when setting up the coil has no difficulty in connecting them with their proper terminals on the primary coil. It is absolutely essential that the four primary layers should be so connected that current travels in the same direction in all of them. For instance, if they were joined up in such a way that the primary current travelled from left to right in two of them and from right to left in the other two, there would be two distinct sets of inductive impulses in the secondary coil which would neutralise each other, and so there would be absolutely no available current at all generated in the secondary winding. It is necessary to emphasise this point because the *simplest* way to connect the primary layers in series —by means of short wires joining up some of the terminals at each end—is the *wrong* way, and would result in the contradictory condition of affairs just described.

### The Working of an Induction Coil.

An induction coil is really a form of step-up transformer in which the inducing current consists of a rapidly interrupted continuous current instead of the alternating current which is used to work a static transformer proper.

When an interrupted continuous current from some source of electricity, such as the house mains, is sent through the primary winding of an induction coil, an entirely fresh current of very high voltage is generated in the secondary winding, and this can be taken off from the discharging pillars which are connected with the beginning and end respectively of the secondary coil. Let us suppose that current is to be sent through the primary coil in a direction from left to right. Firstly, at the instant this current is switched on a fresh current is momentarily generated in the secondary coil and travels along it from right to left; secondly, while current is flowing steadily in the primary there is no current generated in the secondary coil; thirdly, as soon as the primary current is switched off another current is momentarily generated in the secondary coil which travels from right to left—*i.e.*, in the same direction as that in which the primary current had been flowing.

The primary current is said to be "made" when it is switched on and "broken" when it is switched off—hence the currents generated in the secondary coil are known as "make" and "break" currents respectively.

The above phenomena are due to the fact that a magnetic field is set up when current circulates in a primary wire surrounding an iron core, and this magnetic field permeates and has an inductive influence upon every turn of wire in the secondary coil. It is the appearance and disappearance of the magnetic field which induces current in the secondary coil, and the more suddenly the field can be brought to its full strength and made to disappear the stronger will be the current in the secondary coil at make and break respectively.

Obviously, since current is only generated in the secondary wire at the instant when the primary circuit is either made or broken, the more frequently (within certain limits) we can make and break this circuit the more current shall we obtain from the secondary coil; hence some form of mechanical interrupter must be used which will switch on and off the primary current from 50 to 1,000 times per second.

The discharging pillars are fixed as far apart as possible on the secondary coil, one of them usually terminating in a flat plate which is a fixture, while the other supports a sliding rod which can be approximated to or withdrawn from the plate at will. If no external circuit, such as an *x*-ray tube, be provided for the secondary current when the coil is working, the voltage between the discharging pillars is so high that the current will jump across a considerable air-gap to complete the circuit, forming a shower of sparks in the process. The tremendous voltage which these coils will generate can be appreciated when it is stated that a pressure of about 15,000 volts is required to overcome the resistance of an air-gap of one inch, and that some of the coils used in *x*-ray work will discharge sparks across air-gaps of 24 inches or more.

At present, induction coils are classified according to the distance they are capable of sending sparks through the air between their discharging pillars. When the discharging-rods are separated beyond the sparking power of a coil some current still passes between them in the form of a beautiful "brush-discharge" which is plainly visible in a darkened room.

The current induced in the secondary coil is, of course, alternating; that is to say, the discharging pillars become alternately negative and positive as the current is made and broken. The current

* Previous articles appeared on Nov. 11 and 25, Dec. 9 and 30, Jan. 13 and 27, Feb. 17, March 9 and 30.

generated at "make," however, is much weaker than that at "break"; hence we call the pillar which is positive at "break" the + terminal or *anode* of the coil, and the other pillar the − terminal or *cathode*, and we regard the current in the secondary circuit as flowing from the anode, through the external circuit, back to the cathode. For $x$-ray work it is essential to send current through the tube in one direction only: hence it is a fortunate occurrence that the current at make is, comparatively speaking, so weak that most of it can be suppressed. It is this "reverse" current at make which is the bugbear of all radiographers, for it tends to spoil the tube before its time and to blur the outline of the pictures. The more efficiently a coil is constructed the less reverse current does it produce, but even the best coils generate some, and various extrinsic devices are now adopted by different workers to suppress it. The factor inherent in the coil itself which keeps down the reverse current in the secondary (or, in other words, which renders the break current so much stronger than the make current) is known as *self-induction*.

### Self-Induction in the Primary Coil.

Quite apart from the currents produced in the secondary coil, every turn of wire in the primary winding acts inductively upon every other turn. Consequently, each time the current in the primary circuit is made, a momentary current of fairly high voltage is induced in the primary coil and flows in a direction contrary to that of the ordinary primary current. For the same reason, each time the primary circuit is broken another momentary current is induced in the primary coil, but this flows in the same direction as the ordinary primary current. These extra currents are known as currents of "self-induction" because they are produced as a result of the influence which the primary coil exerts upon itself.

We have seen that the more suddenly the magnetic field reaches its maximum strength the greater will be the current induced in the secondary coil at make, and since it is this make current which we wish to suppress, it will be an advantage if we can retard the primary current in such a way that it reaches its maximum very gradually. Now this is exactly what the self-induced current, at make, does, for it acts as a resistance to the sudden flow of the normal primary current; consequently the magnetic field reaches its maximum comparatively slowly and the current induced in the secondary coil at *make* is very markedly *weakened*. But the self-induced current helps to *strengthen* the secondary current at *break* because, with the aid of the condenser, it can be utilised to make the disappearance of the magnetic field in the primary very sudden and complete.

### The Condenser.

The two sides of a condenser may be regarded as the two coatings of a Leyden jar; they are connected with opposite sides of the interrupter and act as a reservoir for the self-induced current. At the moment of breaking the primary circuit self-induced current rushes into the condenser and charges it to a higher voltage than that of the street mains. It then discharges itself through the primary coil, and in doing so helps suddenly to damp out the magnetic field with the result explained above. But the condenser has another important function: the self-induced current, being of comparatively high voltage, tends to produce sparks between the contacts of the interrupter which would be bad for the interrupter and would prolong the primary current and consequently the magnetic field, but if the capacity of the condenser be suitably chosen it will retain all the self-induced current for a sufficient length of time to prevent any sparking in the interrupter.

### General Considerations.

Although the above sequence of events has taken some time to analyse, it must be remembered that everything takes place at least fifty times per second, and much oftener if a rapid interrupter be used.

Some idea of the stimulus which coil-making has received since the introduction of the $x$-rays may be gathered from the fact that with the coils of ten years ago the make current was often as high as 30 per cent. of the break current, whereas in the present-day coils the make current can be almost totally suppressed.

I have spent a great deal of time over technicalities; my justification must be that without a thorough knowledge of the anatomy and physiology of an induction coil it is impossible for anyone to do uniform $x$-ray work.

It has been suggested that it would be more satisfactory to classify coils according to the number of milliampères they are capable of discharging across a standard air-gap which might be conveniently fixed at 8 inches. This method takes into consideration both quantity and pressure, whereas the spark-length method only takes into consideration the pressure (voltage) which the coil is capable of generating. For instance, with a modern coil the writer has obtained a current of 25 milliampères through an air-gap of 8 inches, whereas with a coil he used five years ago he could only obtain four milliampères through the same distance; yet both were nominally 12-inch coils, and both would certainly send a spark through a 12-inch air-gap, but in the one instance the spark was thin and blue, while in the other it was thick and yellow.

In purchasing a coil the interrupter should always be obtained from the same makers, so that it may be perfectly adapted to the condenser and self-induction of the coil. The makers should be asked to furnish a statement as to the number of milliampères (measured with a d'Arsonval type of meter) it will send through a spark-gap of eight inches and as to how many ampères of current are consumed in the primary circuit to produce this output. The latter will afford a valuable indication of the amount of self-induction in the primary coil. The old coils had a low self-induction, consumed a large amount of current, and furnished a very poor output.

(*To be continued.*)

